# How chip size impacts steam pretreatment effectiveness for biological conversion of poplar wood into fermentable sugars

**DOI:** 10.1186/s13068-015-0373-1

**Published:** 2015-12-09

**Authors:** Jaclyn D. DeMartini, Marcus Foston, Xianzhi Meng, Seokwon Jung, Rajeev Kumar, Arthur J. Ragauskas, Charles E. Wyman

**Affiliations:** Department of Chemical and Environmental Engineering, University of California, Riverside, Riverside, CA 92507 USA; Center for Environmental Research and Technology, Bourns College of Engineering, University of California, Riverside, 1084 Columbia Ave, Riverside, CA 92507 USA; BESC BioEnergy Science Center, Oak Ridge National Laboratory, Oak Ridge, TN 37831 USA; DuPont Industrial Biosciences, 925 Page Mill Road, Palo Alto, CA 94303 USA; School of Chemistry and Biochemistry, Institute of Paper Science and Technology, Georgia Institute of Technology, 500 10th St., Atlanta, GA 30332 USA; Department of Energy, Environmental and Chemical Engineering, Washington University, 1 Brookings Drive, Saint Louis, MO 63130 USA; Joint Institute for Biological Sciences, Biosciences Division, Oak Ridge National Laboratory, Oak Ridge, TN 37831 USA; Department of Chemical and Biomolecular Engineering, Center for Renewable Carbon, University of Tennessee, Knoxville, TN 37996–2200 USA; Department of Forestry, Wildlife, and Fisheries, Center for Renewable Carbon, University of Tennessee, Knoxville, TN 37996–2200 USA

**Keywords:** Biofuels, Enzymes, Digestibility, Woody biomass, Pretreatment, Wood chip, Particle size

## Abstract

**Background:**

Woody biomass is highly recalcitrant to enzymatic sugar release and often requires significant size reduction and severe pretreatments to achieve economically viable sugar yields in biological production of sustainable fuels and chemicals. However, because mechanical size reduction of woody biomass can consume significant amounts of energy, it is desirable to minimize size reduction and instead pretreat larger wood chips prior to biological conversion. To date, however, most laboratory research has been performed on materials that are significantly smaller than applicable in a commercial setting. As a result, there is a limited understanding of the effects that larger biomass particle size has on the effectiveness of steam explosion pretreatment and subsequent enzymatic hydrolysis of wood chips.

**Results:**

To address these concerns, novel downscaled analysis and high throughput pretreatment and hydrolysis (HTPH) were applied to examine whether differences exist in the composition and digestibility within a single pretreated wood chip due to heterogeneous pretreatment across its thickness. Heat transfer modeling, Simons’ stain testing, magnetic resonance imaging (MRI), and scanning electron microscopy (SEM) were applied to probe the effects of pretreatment within and between pretreated wood samples to shed light on potential causes of variation, pointing to enzyme accessibility (i.e., pore size) distribution being a key factor dictating enzyme digestibility in these samples. Application of these techniques demonstrated that the effectiveness of pretreatment of *Populus tremuloides* can vary substantially over the chip thickness at short pretreatment times, resulting in spatial digestibility effects and overall lower sugar yields in subsequent enzymatic hydrolysis.

**Conclusions:**

These results indicate that rapid decompression pretreatments (e.g., steam explosion) that specifically alter accessibility at lower temperature conditions are well suited for larger wood chips due to the non-uniformity in temperature and digestibility profiles that can result from high temperature and short pretreatment times. Furthermore, this study also demonstrated that wood chips were hydrated primarily through the natural pore structure during pretreatment, suggesting that preserving the natural grain and transport systems in wood during storage and chipping processes could likely promote pretreatment efficacy and uniformity.

## Background

Woody biomass represents an important source of lignocellulosic biomass for sustainable production of organic chemicals and liquid fuels, with up to 142 million dry tons of sustainably sourced forest biomass and wood waste available in 2012 [1]. This amount has the potential to increase dramatically through future use of dedicated woody bioenergy crops, with between 100 and 300 million dry tons estimated to be produced annually by 2030 [1]. However, woody biomass is recalcitrant to enzymatic sugar release and thus often requires significant size reduction and severe pretreatments to achieve economically viable product yields. Due to its large size and high density, mechanical size reduction of wood has been estimated to consume between 5 and 10 times more energy than required for agricultural residues [2]. The use of wood chips that are larger in size would result in substantially reduced energy requirements for milling, translating into lower conversion costs.

Most laboratory research has utilized small wood sizes (typically <2 mm) to accommodate smaller pretreatment and enzymatic hydrolysis reactors and to differentiate reaction kinetics from heat or mass transfer effects. Although this approach is appropriate for such purposes, milling to small particle sizes is unlikely to be desirable in an industrial setting, and there remains a gap in understanding of how pretreatment affects the digestibility of larger wood chips. To better understand whether the effectiveness of steam explosion pretreatment is limited in an industrially relevant sized wood chip, we sought to address the following questions:

1. Does composition and/or digestibility vary across the thickness of an industrially sized wood chip after pretreatment?
2. Does pretreatment render the exterior of a wood chip more digestible than the interior and why?
3. How does pretreatment efficacy for an industrially sized wood chip vary for different pretreatment times?

As summarized by Vidal Jr. et al. [3], a number of previous studies have provided information relevant to these questions by examining the effect of particle size in various pretreatment regimes. Although these studies generally tested particle sizes <12 mm, the majority of research on steam explosion of woody biomass reported that larger sized materials either exhibited similar [4] or higher glucose yields in subsequent enzymatic hydrolysis [5, 6] compared to smaller sized particles. These studies suggested that chip size, at least up to 12 mm, did not limit effective pretreatment in terms of preparing biomass for enzymatic digestion. However, little work has been done looking at larger wood chips to examine whether differences exist in the composition and digestibility within a single pretreated wood chip due to heterogeneous pretreatment across its thickness. This limitation is due in large part to the significant amounts of material that are typically required for analysis of biomass composition and enzymatic sugar release.

To overcome this limitation, we applied downscaled methods we developed at the University of California Riverside [7, 8] to study potential variations in both composition and sugar release in pretreatment and subsequent enzymatic hydrolysis of sub-sections (or slices) taken across the thickness of a pretreated wood chip measuring 50.8 × 38.1 × 12.7 mm in length, width, and thickness, respectively. Heat transfer modeling, Simons’ stain testing, magnetic resonance imaging (MRI), and scanning electron microscopy (SEM) were applied to shed light on potential causes of variations in sugar yields within and between pretreated wood.

## Results and discussion

Cell wall analysis (i.e., composition and Simons’ stain testing) and enzymatic digestibility assays were applied to milled wood, as well as spatially distinct sub-sections of both unpretreated and steam-pretreated aspen wood chips. This study provides information relevant to understanding and optimizing the pretreatment of large wood chips, including the potential effects that heat and mass transfer have on the spatial heterogeneity of pretreatment across the thickness of a wood chip. This study does not report on the enzymatic sugar yields of intact wood chips, but instead applied enzymatic digestibility testing (among other tools) to each chip section to understand the spatial heterogeneity of pretreatment in large biomass particles.

### Composition

The composition of the raw unpretreated aspen wood was measured to be 51.0 % glucan, 22.3 % xylan, and 21.0 % acid insoluble residue (AcIR). The AcIR measurement includes both lignin and acid insoluble ash so it typically provides an approximate measure of the Klason lignin content. However, since the whole ash content of this batch of *Populus tremuloides* wood was low, <4 % [9], the AcIR also provided a good estimate of Klason lignin content in this case.

**Fig. 1:**
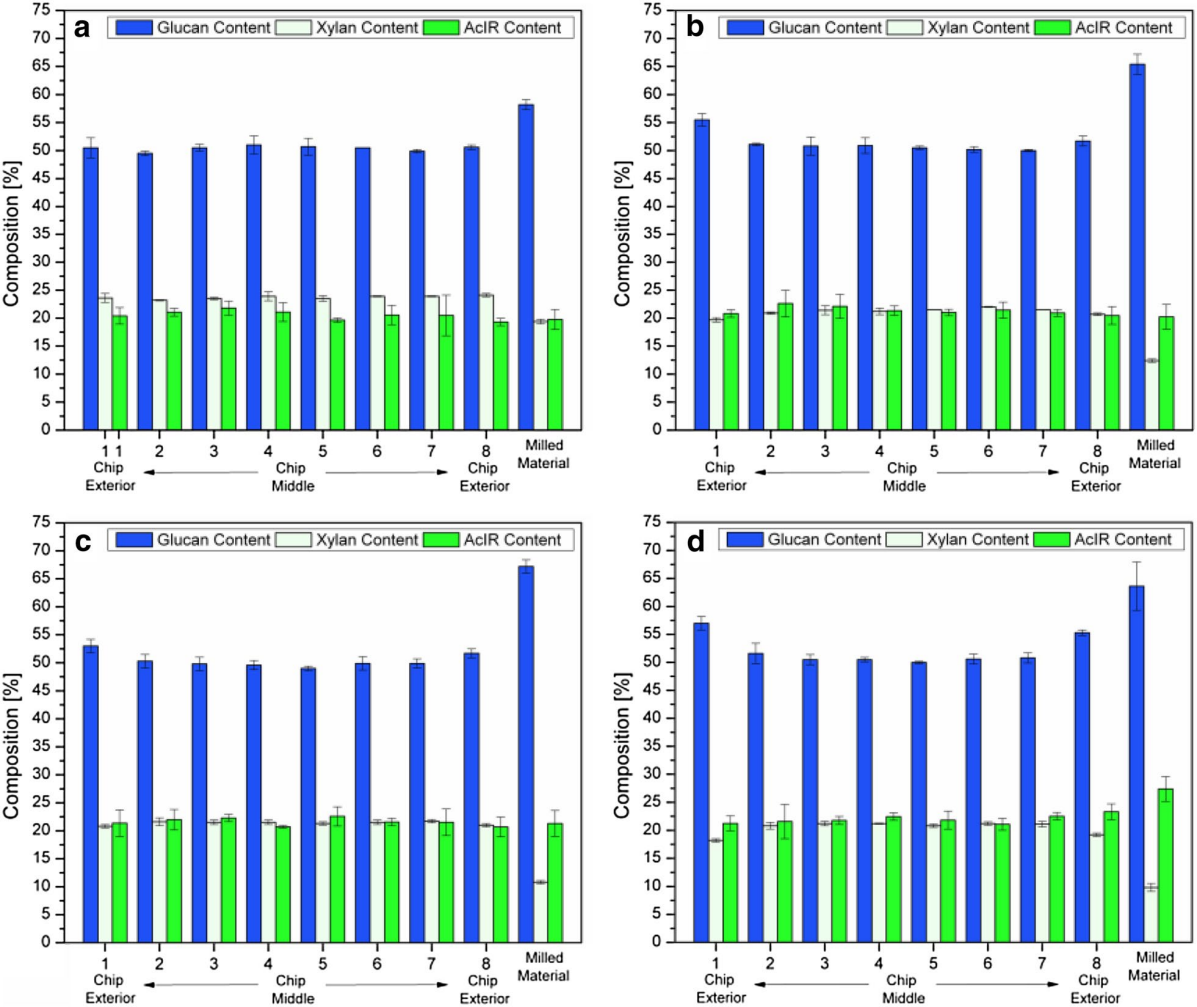
Compositional analysis of aspen wood chips and milled aspen wood pretreated at 180 °C for 4 (**a**), 8 (**b**), 12 (**c**), and 18 (**d**) minutes. Glucan, xylan, and AcIR contents of the steam-pretreated milled aspen are shown on the far *right*, while chip sub-samples are shown to the *left* and labeled 1–8 to represent the 8 layers into which the pretreated wood chips were sectioned, with 1 and 8 being exterior layers, and 2–7 being interior. Downscaled compositional analysis was performed in triplicate, with *error bars* representing the corresponding standard deviation

Figure 1 displays the glucan, xylan, and AcIR contents of each of the four pretreated wood chips (180 °C for 4, 8, 12, and 18 min) as a function of sub-section location across the chips’ thickness (which measures 12.7 mm). Additionally, the composition of wood that was milled prior to pretreatment is also shown at the far right end of the x-axis in each subplot. In comparing compositions across the thickness of a single pretreated chip, preliminary assessment revealed no striking differences in carbohydrate or AcIR content. Indeed, for the 4-min pretreated chip, compositions of the exterior samples (1 and 8) were not significantly different from those of the interior samples (2 through 7) of the same chip. However, the 8, 12, and 18-min pretreated wood chips (Fig. 1b–d) revealed slight differences in glucan and xylan content between the interior and exterior sub-samples that were statistically significant (*p* < 0.001). Thus, in wood chips pretreated for these longer times, the glucan contents of exterior sub-samples 1 and 8 were slightly higher than that of interior sub-samples 2 through 7, while the xylan content followed the opposite trend, with lower xylan content on the exterior of the chip. These results clearly demonstrate compositional variability within industrially sized wood chips pretreated for longer reaction times (>4 min), with the exterior surfaces of the wood chips showing signs of being exposed to a more severe pretreatment.

An important observation to keep in mind is that in general, the glucan and AcIR contents increased, while the xylan contents dropped with increasing pretreatment time (Fig. 1a–d). The average glucan, xylan, and AcIR contents of the 4-min pretreated chip were 50.6, 23.8, and 20.6 %, respectively, whereas the same measurements were 52.0, 20.5, and 22.0 % for the 18-min pretreated chip. These changes were even more pronounced in the milled wood in which the 4-min pretreated material had a composition of 58.2 % glucan, 19.4 % xylan, and 19.8 % AcIR, while the 18-min pretreated milled material had a composition of 63.9 % glucan, 9.8 % xylan, and 27.4 % AcIR. These trends reflect both increased hemicellulose solubilization during water-only steam explosion with longer pretreatment time and greater sensitivity of milled versus chipped wood to increasing pretreatment severity.

### Sugar yields

**Fig. 2:**
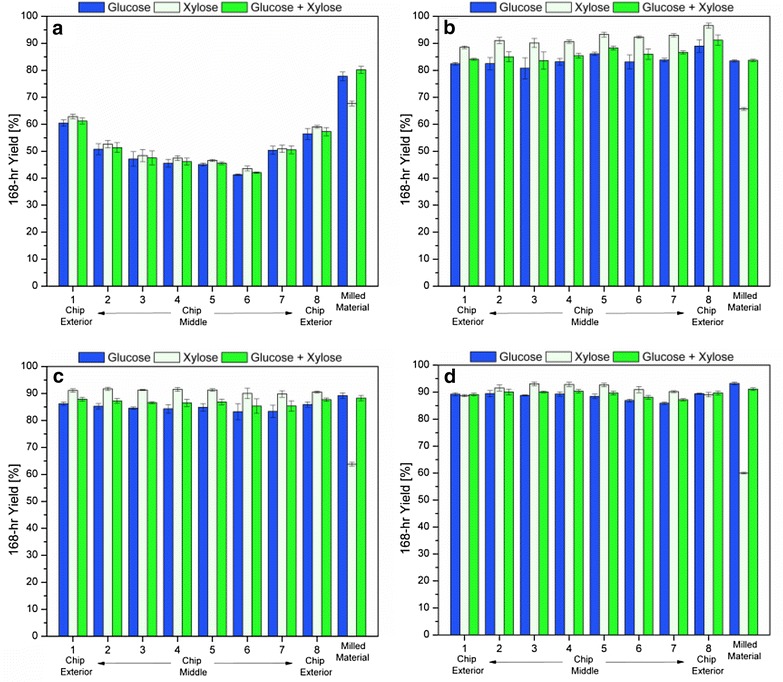
168-h glucose, xylose, and glucose plus xylose yields from enzymatic hydrolysis of each section of pretreated aspen wood chips, as well as milled pretreated aspen. Shown are results from chips and milled wood pretreated at 180 °C for 4 (**a**), 8 (**b**), 12 (**c**), and 18 min (**d**). Yields reflect the amount of each sugar released in enzymatic hydrolysis as a percent of the maximum available of that type of sugar left in the pretreated biomass, as measured by compositional analysis. *Error bars* the standard deviation of triplicate runs

We next sought to measure how the enzymatic digestibility of the pretreated chips changed with pretreatment time and distance from the surface of the chip. Figure 2 plots the 168-h glucose, xylose, and glucose + xylose (“total sugar”) yields from enzymatic hydrolysis as a function of sub-section location across the thickness of each of the four pretreated wood chips. Additionally, the sugar yields are also shown at the far right end of the x-axis for wood that was milled prior to pretreatment. Yields reflect the amount of sugar released in enzymatic hydrolysis as a percent of the total amount of sugar available in the pretreated biomass.

Figure 2 demonstrates that there were no clear trends in glucose or xylose yields across the chips’ thickness for the 8, 12, or 18-min pretreatment conditions. In particular, glucose and xylose yields at the chips’ exteriors were not higher than those of the interiors, suggesting that a 180 °C pretreatment for 8 min or greater did not result in significant spatial effects. However, there was a clear trend of decreasing glucose and xylose yields from the exterior to interior sections for the 4-min pretreated chip, suggesting that the shortest pretreatment time tested resulted in overall lower sugar yields, as well as digestibility spatial effects across the chip’s thickness. For this particular chip, the glucose yields dropped from an average of 58.4 % for the exterior samples to 45.3 % for the innermost two samples. Similarly, the average difference in xylose yield was 60.1 versus 47.0 % for the exteriors and interiors, respectively.

It is also important to note that chips pretreated for longer times generally exhibited higher yields for all sub-samples, as expected. There was a steep drop off in sugar yields for the wood chip pretreated for the shortest time (Fig. 2a), for which the average glucose yield was only about 50 %, as compared to average glucose yields of between 83 and 89 % achieved after 8–18 min pretreatment of the chips (Fig. 2b–d).

In contrast to these results for wood chips, the yields were similar for all pretreatment times tested on the milled materials. As such, the 168-h enzyme treatment resulted in glucose yields from 84.5 % for the shortest 4-min pretreatment time to 94.0 % for the 18-min pretreated milled wood. Thus, final glucose yields for enzymatic hydrolysis were significantly higher for the 4-min pretreated milled wood than they were for the 4-min pretreated wood chip. However, glucose yields were very similar for both chips and milled wood for longer pretreatment times.

### Investigating performance and variability

Based on the variability in enzymatic saccharification results across a chip’s thickness, as well as between chips and milled material, we employed heat transfer modeling, Simons’ stain testing, magnetic resonance imaging (MRI), and scanning electron microscopy (SEM) with the aim of answering the following questions:

1. Why was the 4-min pretreated chip the only one that exhibited significant digestibility differences across its thickness?
2. Are there structural and/or chemical features that caused differences in digestibility for the 4-min pretreated chip?
3. Why did the milled material exhibit significantly higher digestibility than the corresponding chip only for the 4-min pretreatment, and conversely, why were the final glucose yields similar for pretreated chips and the milled materials for application of 8, 12, and 18-min pretreatment times?

#### Heat conduction modeling

Heat transfer throughout a wood chip during pretreatment was modeled to estimate whether this was a factor in limiting effective pretreatment across an entire chip thickness. To this end, a solution for two-dimensional heat conduction through a rectangular cross-section was applied [10]. Because the series solution converges quickly, application of only the first seven terms was needed to predict the temperature profile across the chip’s thickness [11], as shown by Eq. 1,

1 $$\begin{aligned} T & = T_{S} + \left( {T_{0} - T_{S} } \right)\left( {\frac{{16}}{{\pi ^{2} }}} \right) \\ & \quad \times \left\{ {\sin \left( {\frac{{\pi x}}{a}} \right)\sin \left( {\frac{{\pi y}}{b}} \right)\exp \left[ { - \pi ^{2} t\left( {\frac{{\alpha _{x} }}{{a^{2} }} + \frac{{\alpha _{y} }}{{b^{2} }}} \right)} \right] + ~\left( {\frac{1}{3}} \right)\sin \left( {\frac{{3\pi x}}{a}} \right)\sin \left( {\frac{{\pi y}}{b}} \right)\exp } \right. \\ & \quad \quad \times \left[ { - \pi ^{2} t\left( {\frac{{9\alpha _{x} }}{{a^{2} }} + \frac{{\alpha _{y} }}{{b^{2} }}} \right)} \right] + ~\left( {\frac{1}{3}} \right)\sin \left( {\frac{{\pi x}}{a}} \right)\sin \left( {\frac{{3\pi y}}{b}} \right)\exp \left[ { - \pi ^{2} t\left( {\frac{{\alpha _{x} }}{{a^{2} }} + \frac{{9\alpha _{y} }}{{b^{2} }}} \right)} \right] \\ & \quad \quad + ~\left( {\frac{1}{5}} \right)\sin \left( {\frac{{5\pi x}}{a}} \right)\sin \left( {\frac{{\pi y}}{b}} \right)\exp \left[ { - \pi ^{2} t\left( {\frac{{25\alpha _{x} }}{{a^{2} }} + \frac{{\alpha _{y} }}{{b^{2} }}} \right)} \right] \\ & \quad \quad + \left( {\frac{1}{5}} \right)\sin \left( {\frac{{\pi x}}{a}} \right)\sin \left( {\frac{{5\pi y}}{b}} \right)\exp \left[ { - \pi ^{2} t\left( {\frac{{\alpha _{x} }}{{a^{2} }} + \frac{{25\alpha _{y} }}{{b^{2} }}} \right)} \right] \\ & \quad \quad + \left( {\frac{1}{7}} \right)\sin \left( {\frac{{7\pi x}}{a}} \right)\sin \left( {\frac{{\pi y}}{b}} \right)\exp \left[ { - \pi ^{2} t\left( {\frac{{49\alpha _{x} }}{{a^{2} }} + \frac{{\alpha _{y} }}{{b^{2} }}} \right)} \right] \\ & \quad \quad \left. { + \left( {\frac{1}{7}} \right)\sin \left( {\frac{{\pi x}}{a}} \right)\sin \left( {\frac{{7\pi y}}{b}} \right)\exp \left[ { - \pi ^{2} t\left( {\frac{{\alpha _{x} }}{{a^{2} }} + \frac{{49\alpha _{y} }}{{b^{2} }}} \right)} \right] + \cdots } \right\}, \\ \end{aligned}$$

where *T*_*s*_ is the surface temperature (that was assumed to be attained immediately), *T*_0_ is the initial temperature, *a* and *b* are the cross-sectional dimensions, *α*_*x*_ and *α*_*y*_ are the thermal diffusivities in the *x* and *y* directions, respectively, and *t* is time in minute. To calculate the temperature at the center of the chip, the following conditions were set: *x* = *a*/2 and *y* = *b*/2. Furthermore, diffusivity in the radial and tangential directions were assumed to be very similar since they are both against the wood grain, and *α*_*x*_ was set equal to *α*_*y*_ [11]. Other parameters used in this study are listed in Table 1. The diffusivity values were obtained from Abasaeed et al. [12], and represent a range of values for conduction in both the radial and longitudinal directions, as well as conduction in the radial direction in hemicellulose-free wood; these values were determined experimentally for the hardwood species southern red oak.

**Table 1:** List of parameters used to model heat transfer through a wood chip

*T*_s_/*T*_ht_/*T*_ctr_	180 °C
*T*_0_/*T*_init_	22 °C
*a*/TH	0.5″ (0.0127 m)
*b*/W	1.25″ (0.03175 m)
*α*_*x*_ = *α*_*y*_	1.27 × 10^−7^ m^2^/s^a^
	1.74 × 10^−7^ m^2^/s^b^
	2.63 × 10^−7^ m^2^/s^c^
*M*	22.26 %
*G*	0.6505^d^
Constants: *a*/*b*/*c*/*d*	60.44/−3.032/3.080/−0.2662
Constants: *e*/*f*/*g*/*h*	1.720/−0.2560/−0.0945/0.2156

^a^For conduction in radial direction (against grain)

^b^For conduction in radial direction (against grain) in hemicellulose-free wood

^c^For conduction in longitudinal direction (with grain)

^d^The specific gravity was taken to be the average of 0.546 and 0.755 from reports in literature

**Fig. 3:**
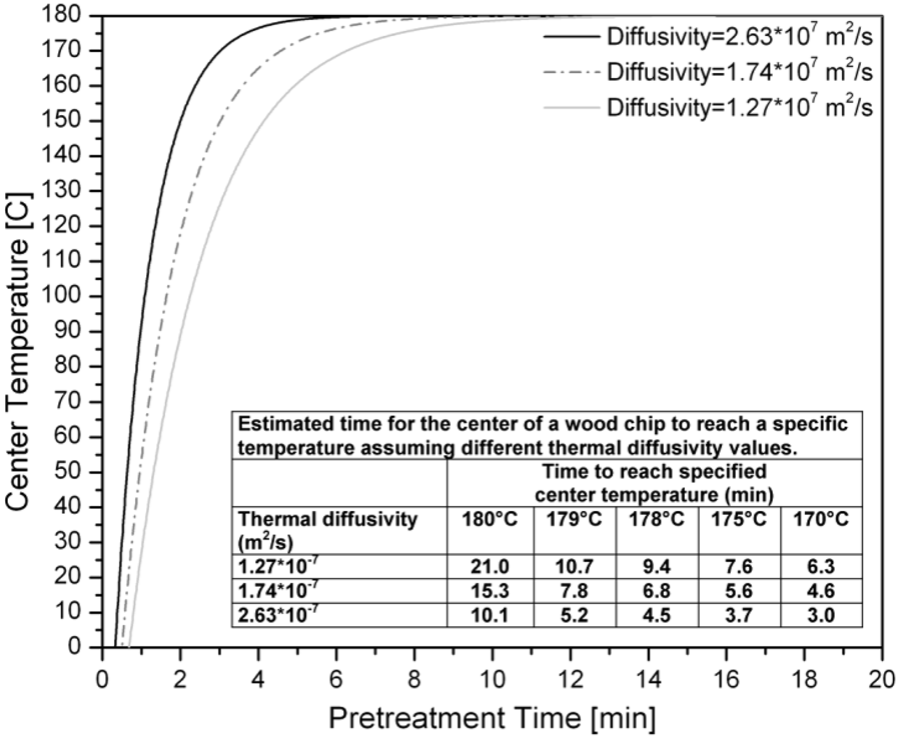
Predicted temperature at the center of a wood chip with dimensions used in this study versus pretreatment time at 180 °C based on a solution to two-dimensional heat conduction through a rectangular cross-section [10, 11]. Temperature profiles are given for 3 thermal diffusivities to represent a range of possible values that depend on grain direction, wood density, and hemicellulose content (Abasaeed et al. [12]). The inset table displays the numerical values of the estimated times it takes for the center of the wood chip to reach a specific temperature for each diffusivity

Based on the analysis described above, the temperature at the center of the wood chip was plotted versus pretreatment time in Fig. 3 for the three different assumed thermal diffusivity values. The results show that the temperature at the center of the wood chip increased rapidly during the first couple of minutes of pretreatment and then asymptotically approached the target temperature of 180 °C. The inset table in Fig. 3 summarizes the time it took to reach a specific center temperature for the different thermal diffusivity values. As such, this model predicts that it would take between 3.7 and 7.6 min for the center of the chip to reach within 5 °C of the target temperature, depending on the wood thermal diffusivity assumed.

An additional model was also applied to provide a second estimate of the heating time. The analysis by Simpson [13] is based on a multiple regression analysis in which heating times for a large combination of variables (including chip size, temperature, and wood-specific gravity) were calculated using heat conduction equations and then fitted to the regression model in Eq. 2.

2 $${\text{T = a}}\left( {{\text{T}}_{{{\text{ht}}}} } \right)^{{\text{b}}} \left( {{\text{T}}_{{{\text{ctr}}}} } \right)^{{\text{c}}} \left( {{\text{T}}_{{{\text{init}}}} } \right)^{{\text{d}}} \left( {{\text{TH}}} \right)^{{\text{e}}} {\text{W}}^{{\text{f}}} {\text{M}}^{{\text{g}}} {\text{G}}^{{\text{h}}}$$

where T_ht_ is the heating temperature, T_ctr_ is the target center temperature, T_init_ is the initial temperature, TH is the chip thickness, W is the chip width, M is the chip moisture content (%), G is the specific gravity of the wood chip, and *a*–*h* are pre-determined regression coefficients. The inputs applied in this model are also listed in Table 1. As such, this model estimated that it would take approximately 4.8 min for the center of the wood chip to reach its target temperature. These results are in line with those obtained from the previous model displayed in Fig. 3: both suggested that it should take about 5 min for the center of the wood chip to reach close to the target temperature. Thus, it seems probable that the 4-min pretreatment tested in this study was insufficient to attain the target temperature uniformly throughout the thickness of the wood chip. However, the model also predicted that the entire thickness of wood chip should reach the target temperature, or very close to it, following pretreatment for 8–18 min, which is in line with the more uniform yields in Fig. 2b–d. These results suggest that chip size has an important effect on the reaction time needed to achieve effective and uniform pretreatment on a wood chip at industrial scale due to heat transfer effects.

While a pretreatment reaction temperature of 180 °C was studied in this paper, these models can also be used to evaluate other pretreatment conditions as well. For example, if the temperature were raised from 180 to 200 °C, as is frequently done for hydrothermal pretreatments, the optimum reaction time needed would drop by a factor of about four based on the pretreatment severity parameter [14]. The models discussed above suggest that heat transfer concerns and non-uniformity throughout a wood chip would become more of an issue under these shorter pretreatment times. In general, this study suggests that it would be wise to avoid extremely high pretreatment temperatures and their corresponding short reaction times since non-uniformity in temperature and yield profiles will become an issue in large wood chips.

#### Composition and enzyme inhibition

Since modeling suggested that pretreatment effectiveness may be impacted by temperature gradients across a wood chip, it was sought to determine what effect this differential heating profile had on substrate characteristics that may account for differing digestibility across the thickness of pretreated wood chips. Specifically, what changes to chemical and structural features may have contributed to lower enzymatic sugar yields for the inner sections of the 4-min pretreated chip compared to the outer sections?

To determine whether differences in the solids composition could account for yield variations across the thickness of the 4-min pretreated chip, 168-h glucose yields were plotted versus xylan and AcIR contents for the various sections (plots not shown). These characteristics were selected because glucose yields have previously been found to be positively correlated with xylan removal, due to increased enzyme accessibility [15, 16]. Glucose yields have also been reported to be inversely related to lignin content (which is by far the largest component of AcIR) due to lignin’s role in restricting enzyme accessibility, as well as non-productive enzyme binding to lignin [17–19]. However, no correlation or trend was observed in this dataset suggesting that neither of these factors could account for the differential sugar yields in the 4-min pretreated chip.

The second factor investigated was whether soluble sugars and oligomers may have influenced compositional analysis results and/or caused enzyme inhibition during hydrolysis. Because compositional analysis and enzymatic hydrolysis employed unwashed pretreated solids, soluble sugars such as xylose or xylooligomers may have resulted in overestimation of the xylan content for some samples, perhaps influencing the sugar yields discussed previously. In addition to potentially influencing composition, differences in the xylooligomer concentration could also result in differences in enzyme inhibition. For example, a higher xylooligomer-to-xylose ratio in the interior chip slices could result in greater enzyme inhibition for these samples [20] and help explain the lower yields observed (Fig. 2a). To test for this possibility, dried samples of the 4-min pretreated chips were washed with hot water to remove soluble sugars. The resulting monomeric and oligomeric sugars were measured in the liquid washates, while the composition and enzymatic hydrolysis were determined for the dried, washed solids. Liquid analysis demonstrated that the soluble sugar content was low in the 4-min pretreated chip: less than 6.5 % of the xylan was left in the form of soluble monomer xylose in the chip after 4 min of pretreatment. Furthermore, post-hydrolysis of the liquid washates revealed an average xylooligomer-to-xylose ratio of 2.0 for two exterior samples (sub-sections 1 and 8), while the average was 1.1 for all six interior samples. The higher level of xylooligomers in the exterior chip sections was the opposite of what was expected, and as a result, does not explain the higher sugar yields seen in these exterior chip slices. Likewise, compositional analysis of the washed solids indicated no glucan or xylan variations across the thickness of the washed pretreated chip samples. Finally, the digestibility of the washed samples did not change significantly from that of the unwashed materials. All of these pieces of evidence point to soluble monomer and oligomeric sugars not being the cause of the digestibility differences in the 4-min pretreated chip.

#### Moisture content and local water mobility: results from MRI

To further probe potential cause(s) of digestibility differences observed within the 4-min pretreated chip, as well as between chips pretreated for longer times, nuclear magnetic resonance imaging (MRI) was applied. Water can be found spatially localized in biomass on cellulose fibril surfaces, within capillaries of lumens, between fibers, and inside lignocellulosic cell wall voids [21–24]. Furthermore, the amount of adsorbed water and strength of association with lignocellulosic substrate has been directly correlated to a combination of recalcitrance relevant characteristics, primarily pore surface area to volume ratio [25–27] and the ultrastructural and chemical states of biomass. As a result, nuclear magnetic resonance (NMR) is routinely utilized to directly monitor the amount of adsorbed water (moisture content) by signal intensity and the proportion of bound to free water protons (pore surface area to volume ratio) by signal relaxation rate. Biomass with less hydrophilicity, e.g., biomass with altered composition (higher lignin content), would absorb less water and have a lower ratio of bound to free water compared to biomass with lower lignin contents. However, because compositional differences among samples were minimal in this study, variation in the amount of adsorbed water, or the proportion of bound to free water protons was not associated with lignin content. On the other hand, biomass with an increased average pore diameter would be expected to absorb more water while displaying a lower ratio of bound to free water. Several published studies applied NMR without spatial information to determine moisture content [28–31] or pore size distributions [24, 27, 32, 33]. Conversely, a variety of other studies have demonstrated the usefulness of MRI as a spatially resolved technique for biomass moisture measurements [34–37]. Recent advances in MRI methodology such as zero echo time (ZTE) imaging, single-point imaging (SPI), and single-point ramped imaging with *T*_1_ enhancement (SPRITE) can reveal images of structures that were previously not discernible [38–41]. In this study, these techniques were applied to spatially monitor proton density of adsorbed water in untreated and pretreated wood chips.

**Fig. 4:**
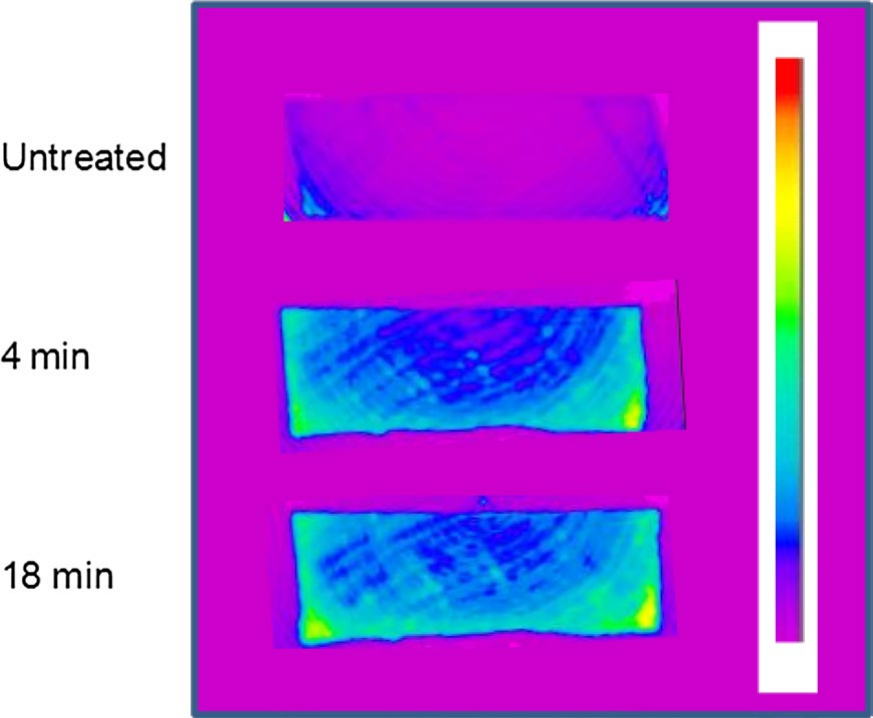
Characteristic ZTE images of cross-sectional slices taken from *z* = 15 mm from the front of the *Populus* wood chips, both untreated and pretreated in a steam gun at 180 °C for 4 and 18 min followed by conditioning at 100 % RH for 14 days. *Red* the highest proton density and water content. The *z* direction corresponds to the chip length and the *x* direction corresponds to chip thickness

**Fig. 5:**
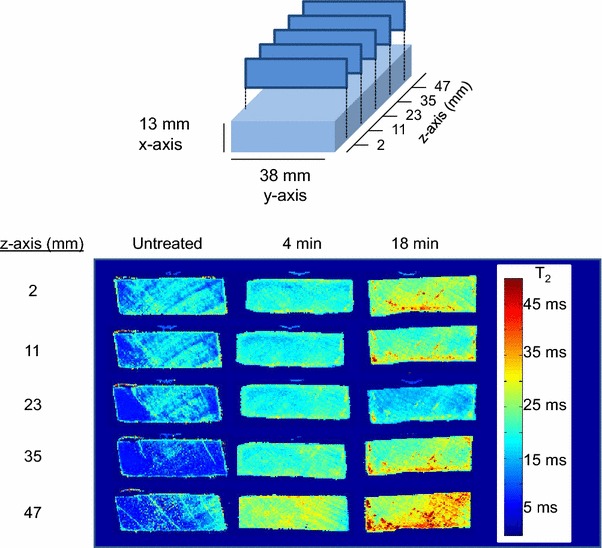
*T*_2_ images resulting from spin echo MRI measurements of *Populus* wood chips, both untreated and pretreated in a steam gun at 180 °C for 4 and 18 min followed by conditioning at 100 % RH for 14 days. *Red* the highest water local mobility. The *z* direction corresponds to the chip length and the *x* direction corresponds to chip thickness

Figure 4 presents the ZTE images for an untreated *Populus* wood chip, as well as chips pretreated at 180 °C for 4 and 18 min. All chips were conditioned at 100 % relative humidity (RH) for 14 days prior to analysis. The colors in the image map indicate the amplitude of the resulting MRI signal or proton density, with red approximately denoting the highest density of water-related protons. The cross-sectional images displayed in Fig. 4 were taken ~15 mm from the edge of the wood chips (or *z* = 15 mm as defined in the illustration of atop Fig. 5), with the exterior chip sub-samples located at the top and bottom of each image, and the interior sub-samples within. High spatial resolution moisture profiles were clearly obtained. The image of the untreated chip shows that some natural variation in moisture content occurred within a wood chip even in the absence of pretreatment. Furthermore, Fig. 4 also demonstrates that the relative moisture content, which can be related to total pore volume, generally increased with pretreatment, with higher proton densities or moisture contents near the outer regions of both pretreated chips. One of the key observations that can be taken from Fig. 4 is the migration of higher proton densities toward the center of the 18-min pretreated chip, whereas migration to the center was very limited in the chip pretreated for only 4 min. This is evidenced by the lighter blue/green colors that can be observed in the natural grain of the wood toward the center of the 18-min chip. There is a noticeably larger section of dark blue and purple colors in the 4-min pretreated chip, indicating regions of lower proton densities toward the center of the chip. These results suggest that differences in moisture content and total pore volume may begin to explain some of the variability observed in saccharification yields across the thickness of the 4-min pretreated chip, whereas this difference was not observed for the longer pretreatment time.

It should be noted that the higher proton densities displayed toward the bottom of the chip images resulted from non-uniform intensity due to susceptibility and non-homogeneous radio frequency receiver/transmission coil and magnetic field profiles. This conclusion was confirmed by the observation of similar artifacts at the bottom of the images when the wood chips were rotated 180° in the height–width or *x*–*y* plane. In addition, alignment of the wood chip with respect to the length or *z* direction of the coil seemed to also significantly affect image intensity along the *z* direction of the image. Although the result of these artifacts was that quantitative spatial interpretation of moisture contents in three dimensions was not possible, all image slices trended towards increased moisture content in the pretreated chips compared to the untreated chip. This result was confirmed with single pulse experiments.

In addition to measuring moisture content, MRI was also utilized to examine the local mobility of adsorbed water within a single chip and among wood chips pretreated for different times by determining the water *T*_2_ time spatial distribution. Spin echo images were recorded for 8 echo times between 7.9 and 63.0 ms over 20 slices, each 1 mm thick, at approximately every 2 mm along the length of the wood chip. Figure 5 shows 5 cross-sectional *T*_2_ images resulting from spin echo MRI experiments on conditioned untreated and pretreated *Populus* wood chips. Unlike the ZTE images, these spin echo MRI experiments were not as sensitive to susceptibility and non-homogeneous radio frequency coil and magnetic field effects, and therefore should provide more reliable spatial information, particularly at image edges. The *T*_2_ values represent the local mobility of absorbed water and thus, the amount of bound versus unbound water within the pores. The ratio of bound to unbound water within the pores can then be related to the surface area to volume ratio. The images clearly showed an increase in *T*_2_ values of absorbed water with pretreatment time, with an average increase of about 20 ms from the untreated to 18-min pretreated chip. This result suggests that a relative increase in the proportion of free water occurred with pretreatment, which in turn, suggests a decrease in the pore surface area to volume ratio. Figure 5 also indicates that water mobility was highly spatially heterogeneous in all chips. The faint yellow observed for the 4-min pretreated chip images indicates higher *T*_2_ values near the perimeter of the chip cross-section, with the exception of slice *z* = 47 mm, which showed higher *T*_2_ values across the entire thickness in general. Low *T*_2_ values (<18 ms) were not observed on the edge of any slices from the 4-min chip, and instead were somewhat localized in the interior. The 18-min sample also showed a similar and perhaps even more heterogeneous spatial distribution of *T*_2_ values across the chip’s thickness; however, unlike the 4-min sample, the spatial effects were less localized, with regions of high mobility (>30 ms) appearing to have migrated more into the center of the 18-min chip’s thickness for all slices except the middle-most slice at *z* = 23 mm.

The trends are consistent with two observations from the digestibility data above: (1) the overall glucose yields from wood chips increased with increasing pretreatment time and (2) the 4-min pretreated chip exhibited digestibility spatial effects across its thickness, whereas spatial effects were not observed for chips pretreated for longer times. These results suggest that higher water mobility, meaning lower surface area to volume ratio, could be positively correlated with better digestibility in these samples. At first glance, it may appear counter intuitive that samples exhibiting higher digestibility would have lower surface area to volume ratios. However, a decreasing ratio of pore surface area to volume (which for a sphere, simplifies to 6 divided by the diameter) is in this case associated with an increase in pore diameter. Thus, wood chips and regions of pretreated chips that exhibited higher digestibility were characterized by pores with relatively larger diameters. These findings suggest that differences in pore size, which can be related to enzyme accessibility, were at least partly responsible for variation in glucose yields observed within and between wood chips. Previous research has clearly demonstrated that enzymatic digestibility of pretreated biomass is directly related to cellulase accessibility, indicating a minimal average cell wall pore diameter of 51 Å is required for enzyme accessibility and significant activity [42]. It would therefore stand to reason that increases in pore surface area in systems with average pore diameters less than 51 Å would do little to increase enzymatic digestibility.

Additionally, a particularly important finding from the MRI studies was that the proton density and mobility of water matched well with the grain of the wood in the untreated wood chip, indicating wood chips were hydrated primarily through the natural pore structure during the initial stages of pretreatment. Thus, axial wood chipping that preserves the natural grain and transport systems in wood could promote pretreatment efficiency and uniformity within an industrially sized chip, an effect also seen in pulping of wood for paper production [43]. Another relevant pulp and paper study indicated that pre-compression of wood before chipping can further open the wood structure and increase specific surface area [44, 45], facilitating penetration and uptake of chemicals and water during pretreatment.

#### Total pore volume and relative accessibility: results from Simons’ stain

It seems intuitive that smaller particle sizes could result in more effective pretreatment due to a higher particle surface area to volume ratio. However, Fig. 2 shows that milled material exhibited higher final glucose yields versus chips for the 4-min pretreatment time only; conversely, the final glucose yields were very similar for chips and milled material for 8, 12, and 18-min pretreatment times.

**Fig. 6:**
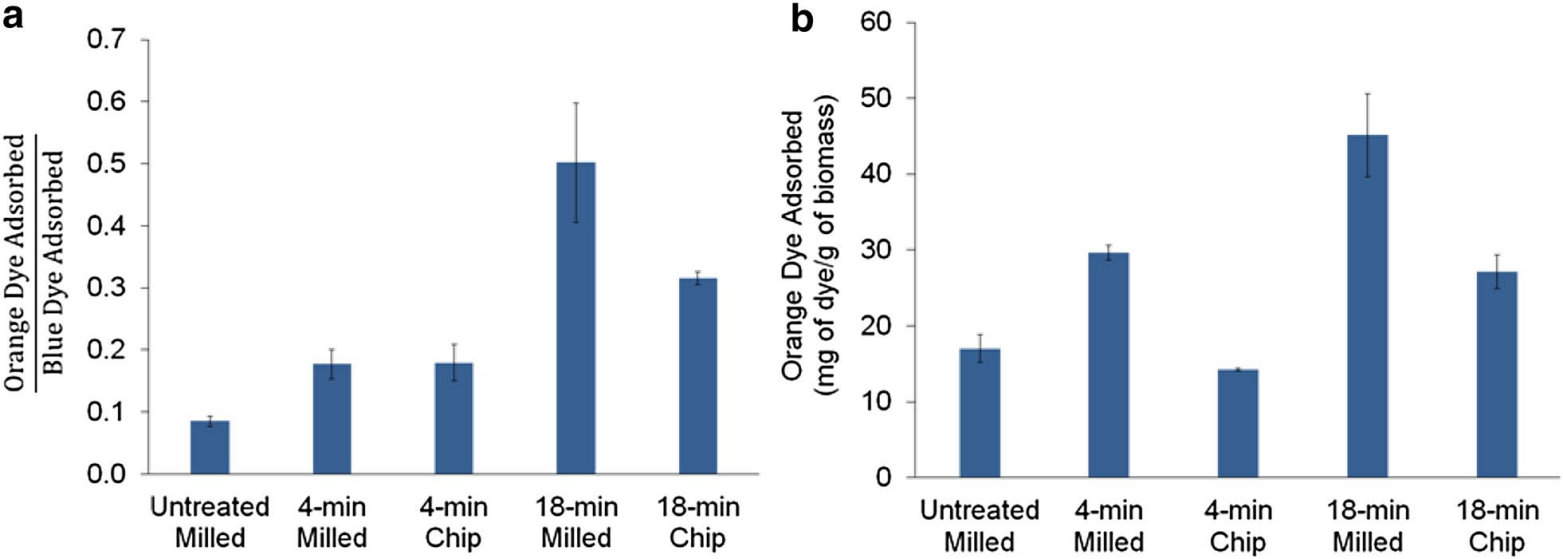
Simons’ stain results for chip and milled materials pretreated for 4 and 18 min reporting the **a** ratio of adsorbed orange dye to adsorbed blue dye and **b** total amount of adsorbed orange dye

To try to address this observation, a Simons’ stain test was performed based on competitive adsorption of two different-sized molecular dyes [46]: an orange polymeric dye with a hydrodynamic diameter between ~6 and 35 nm and smaller blue dye with a hydrodynamic diameter of ~1 nm. This approach can provide insights into the relative enzyme accessibility of biomass samples. As displayed in Fig. 6, Simons’ stain was applied to (1) the 4-min pretreated chip, (2) the 4-min pretreated milled material, (3) the 18-min pretreated chip, and (4) the 18-min pretreated milled material. It should also be noted that the Simons’ stain results for the wood chip material represent an average over the entire thickness of the wood chip (instead of sub-section locations). In comparing the 4-min pretreated chip and corresponding milled material, the Simons’ stain results suggest that the chip and milled wood have similar ratios of adsorbed orange dye to adsorbed blue dye (i.e., the ratio of large to small pores). However, the total adsorbed orange dye (i.e., the amount of surface area associated with larger pores) is greater for the 4-min pretreated milled material versus the corresponding chip. For the longer pretreatment time (18 min), there was a significant increase in both the ratio of adsorbed orange dye to adsorbed blue dye, as well as the total adsorbed orange dye, particularly for the milled material.

The ratio of adsorbed orange dye to adsorbed blue dye and the total adsorbed dye measured by Simons’ stain has been strongly correlated with enzyme accessibility to cellulose and thus enzymatic digestibility [47]. Simons’ stain results here suggest that (1) pretreating the chip for only 4 min did not result in any increase in large pores versus the raw unpretreated wood, (2) accessibility to large pores was higher in the milled material versus the chip, regardless of pretreatment time, and (3) accessibility of the 18-min material was higher than the accessibility of the 4-min material, regardless of wood format (chip vs. milled). Thus, if the trends exhibited for glucose yields in Fig. 2a, d are primarily related to enzyme accessibility, these Simons’ stain results suggest that short pretreatment times may severely limit the production of large pores in wood chips, resulting in poor enzyme accessibility and low sugar yields. However, these results also suggest an accessibility threshold, above which enzymatic digestibility does not depend heavily on further pore size expansion. Thus, further size reduction of woody biomass subjected to long pretreatment times may only have an incremental effect on enzymatic digestibility.

Increased enzyme accessibility resulting from pretreatment (i.e., steam explosion) is typically attributed to solubilization/rearrangement of cell wall polymers and ultrastructure, as well as pore expansion due to the decompression of steam. Based on the compositional changes reported in Fig. 1a, d between the milled and chip materials, as well as between materials pretreated for different times, the increased enzyme accessibility can be at least partially explained by solubilization of cell wall polymers. However, microscopy was also employed to further evaluate the possibility of cell wall polymer rearrangement and ultrastructure disruption that would not be measured by compositional analysis.

#### Ultrastructural changes: results from SEM

**Fig. 7:**
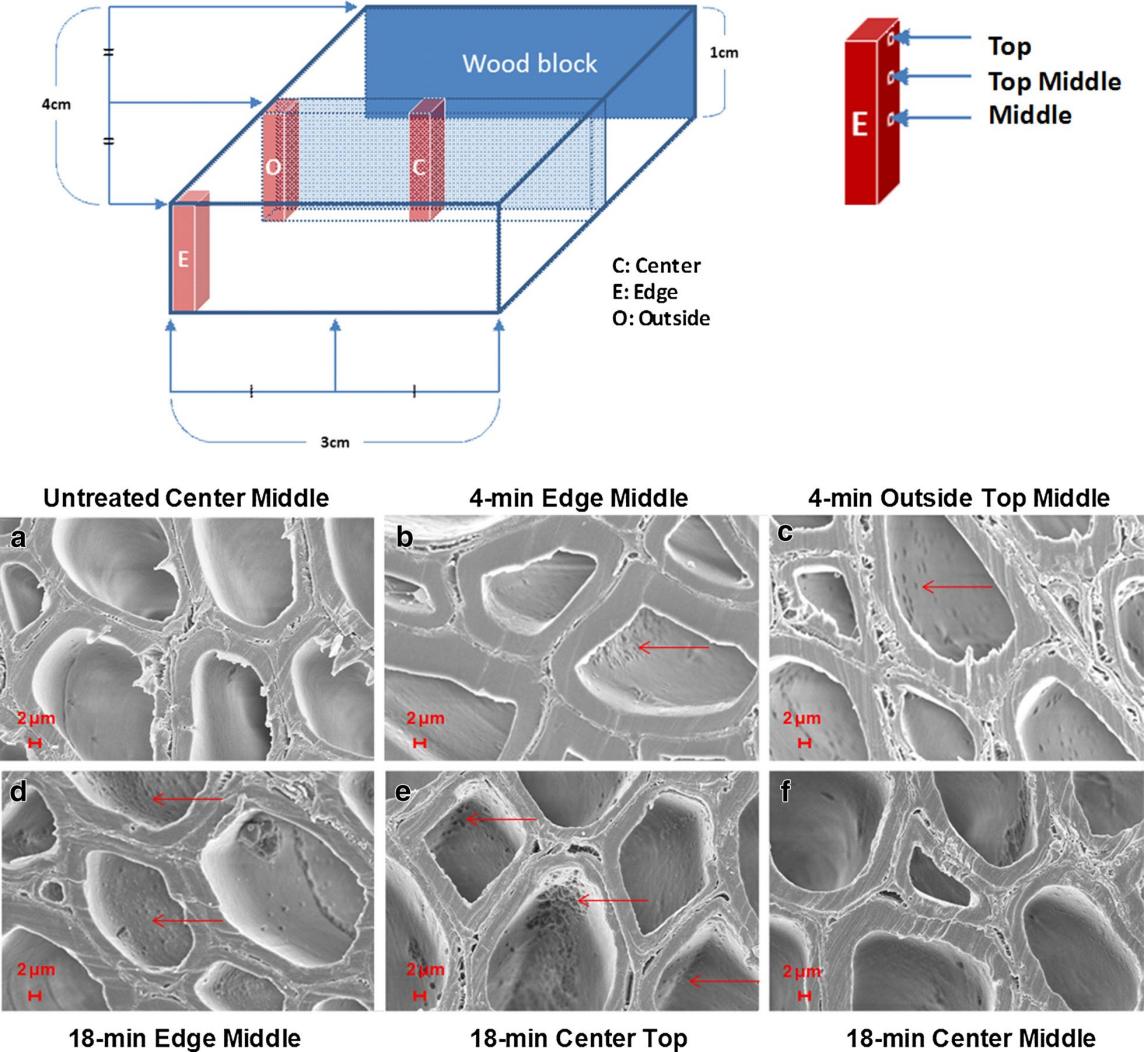
Diagram of the wood block and selected sample positions for morphological analysis by scanning electron microscopy (*top*). Electron micrographs of cross-sections of the wood block (*bottom*): **a** untreated sample at the center middle location, **b** 4-min pretreated sample at the edge middle location, **c** 4-min pretreated sample at the outside *top middle* location, **d** 18-min pretreated sample at the edge *middle* location, **e** 18-min pretreated sample at the *center* *top* location, and **f** 18-min pretreated sample at the *center middle* location. *Red arrows* lignin aggregates. *Scale bar* 2 μm

To further understand how steam explosion pretreatment may affect wood chips, scanning electron microscopy (SEM) was applied to cross-sections from various locations in the untreated, as well as the 4- and 18-min pretreated poplar chip samples, with selected images shown in Fig. 7. The results shown are for three rectangular sized samples cut from the wood chip shown in the diagram at the top of Fig. 7 in red and denoted as edge, outside, and center. Microtome slices were sampled from each rectangular sized chip sub-section at locations again shown in the diagram in Fig. 7 and denoted as top, top middle, and middle. The images, at a resolution of 2 μm (Fig. 7a–f), clearly show that the cell wall vessel elements, secondary cell wall layers, cell corners, and middle lamella remained intact and fairly undisturbed even after pretreatment. Additionally, unlike the untreated wood chip images (Fig. 7a), the images of cross-sections taken from the 4-min pretreated chip displayed what are most likely hemispherical typed lignin aggregates (Fig. 7b, c, denoted by red arrows) seen primarily on the inner portion of the cell walls. This phenomenon of lignin aggregation during dilute acid pretreatment has been well cited for milled material [48]. Furthermore, for the longer 18-min pretreatment time, lignin aggregates in the SEM images (Fig. 7e, f) appear more prevalent, and their shape appears to be more spherical for wood chips than for milled material. While these observations support the solubilization/rearrangement of cell wall polymers and the presumed corresponding increase in enzyme accessibility, SEM images did not reveal any other clear significant structural differences that could further explain the widely different digestibility characteristics between the 4- and 18-min pretreated chips.

## Conclusions

This study shows that efficient and uniform pretreatment is possible for industrially sized poplar wood chips under certain reaction conditions. In particular, materials that were pretreated at 180 °C for 8 min or longer achieved reasonably uniform enzymatic sugar yields across their entire thickness. Conversely, enzymatic digestibility was found to vary substantially within wood chips pretreated for short reaction times (4 min), resulting in digestibility spatial effects and overall lower yields. Based on analysis of the pretreated chips and milled materials, heat transfer modeling suggested that obtaining uniformly high sugar yields requires that the reaction time be sufficient to allow the target pretreatment temperature to be achieved throughout the entire thickness of the wood chip. MRI and Simons’ stain testing both demonstrated that digestibility of the resulting pretreated wood samples could be related to pore size distribution that in turn could be a key factor in dictating enzyme accessibility. These results indicate that rapid decompression pretreatments (e.g., steam explosion) that specifically alter accessibility at lower temperature conditions are well suited for larger wood chips due to the non-uniformity in temperature and digestibility profiles that can result from high temperature and short pretreatments. Additionally, this study also showed that wood chips were hydrated primarily through the natural pore structure during pretreatment, suggesting that preserving the natural grain and transport systems in the wood during chipping would promote pretreatment efficacy and uniformity for biological conversion of industrially sized wood chips.

## Methods

### Biomass samples and material preparation

Trembling Aspen (*Populus tremuloides*) samples were provided by Benchmark International in High Level, Alberta, Canada. Trees were destructively sampled to collect approximately 20–80 mm thick disks, or cross-sections, from individual trees at 0.3 m from the point of germination. The sections were shipped to the University of California Riverside, USA, where they were frozen at 0 °C until use. The cross-section used in this study was from an 80- to 90-year-old tree.

After allowing the cross-section to thaw in a refrigerator, chips were made by a hand chisel. All chips were taken from the outer portion of the section, or the mature wood, to minimize variability within a single chip. Chips had the following dimensions: 50.8 × 38.1 × 12.7 mm in length, width, and thickness, respectively. The moisture content of the chips was approximately 22 %. Additional material was taken from the cross-section and milled through a 20-mesh screen (<0.85 mm) and allowed to air dry until the moisture content reached about 6 %.

### Pretreatment

For steam explosion pretreatment without the addition of any external catalysts, a single chip was placed into a woven metal mesh (8 × 8 mesh particle-sifting woven 316 stainless steel 0.635 mm diameter wire cloth, McMaster Carr, CA, USA) basket, which was then suspended in a 4 L Hastelloy steam reactor in the fashion of a tea bag. Steam for pretreatment was provided by a Fulton steam boiler (FB-075-L, Fulton Companies, Pulaski, NY, USA), which was controlled by setting the boiler pressure to the saturated steam pressure corresponding to the target temperature of 180 °C. Pretreatments were performed at 180 °C for 4, 8, 12, or 18 min. At the end of the reaction time, the temperature and pressure was suddenly dropped by opening a valve at the bottom of the vessel, during which all pretreatment liquid was discharged and not collected for analysis. Afterwards the chip, which remained intact, was removed from the steam reactor for further analysis.

For pretreatment of the milled material, about 4.50 g of material was loaded into a cylindrical catalyst basket whose surface (except the top and bottom faces) was made of metal that was similar in mesh size to that of the basket used for the chip pretreatment. The catalyst basket was suspended and pretreated in the steam chamber as described above. Due to the explosive nature of the flashing step and possible loss of mass from the catalyst basket, an accurate mass balance could not be performed.

### Material preparation

For the pretreated milled wood, the material was allowed to air dry after pretreatment until the moisture content reached approximately 6 %. Immediately following pretreatment, the pretreated chips were fractioned into eight sub-sections across the chips’ 12.7 mm thickness. Thus, a single pretreated chip produced eight sub-sections that were each 50.8 × 38.1 × 1.59 mm in length, width, and thickness, respectively. 32 samples were produced in total from the four pretreated chips. After sectioning, all chip sub-sections were air-dried for 2 days until the moisture content reached approximately 6 %. These samples were then milled through a 20-mesh screen (<0.85 mm) and collected for further analysis. Separate wood chips were pretreated for MRI studies, and treated as described in Sect. “Magnetic resonance imaging”.

Additionally, some of the milled material from the 4-min pretreated chip sub-samples was also washed to remove any soluble sugars or other components that may have remained with the wood after pretreatment. As such, 15 mg of dried biomass from all 8 chip sub-samples of the 4-min pretreated chip was weighed in duplicate into 1.5 mL glass HPLC vials by a solid and liquid dispensing robotic platform (Core Module Standard Configuration 2 equipped with Sartorius WZA65-CW balance and 10–25 mL biomass-dispensing hoppers, Symyx Technologies, Sunnyvale, CA). 800 μL of deionized (DI) water was then added to all vials. Next, vials were placed into an ultrasonic cleaner and sonicated for 90 min at 50 °C. Afterwards, the vials were centrifuged (Allegra X-15R, Beckman Coulter, Fullerton, CA, USA), and the liquid washates were removed for further analysis. The solids were washed two times with DI water by centrifugation and re-suspension and then allowed to dry at room temperature until the moisture content was <6 %. The dried washed solids were saved for compositional analysis and enzymatic hydrolysis.

### Compositional analysis

Glucan, xylan, and acid insoluble residue (AcIR) contents were determined for the pretreated milled wood and each of the chip sub-sections (as well as the washed solids) with a downscaled wet chemistry compositional analysis coupled with high-performance liquid chromatography (HPLC) and gravimetric methods to allow analysis of the small amounts of materials [8]. The procedure is nearly identical to conventional procedures [49] but uses 100 times less biomass (3 mg versus 300 mg) and can be automated using a solid and liquid dispensing robotics platform (Core Module Standard Configuration 2 equipped with Sartorius WZA65-CW balance and 10–25 mL biomass-dispensing hoppers, Symyx Technologies, Sunnyvale, CA, USA) due to the use of 1.5 mL glass HPLC vials as reactors.

Sugar concentrations were measured by HPLC (Alliance 2695 equipped with 2414 RI detector, Waters, Milford, MA, USA) on an Aminex HPX-87H column (Bio-Rad, Hercules, CA, USA) heated to 65 °C and with 0.005 M sulfuric acid as the eluent (0.6 mL/min flow rate). AcIR contents were determined by gravimetric methods to estimate Klason lignin content. Unlike the conventional method, this downscaled procedure measures total acid insoluble residue including acid insoluble ash. However, due to the low whole ash contents of *Populus tremuloides* [9], the acid insoluble residue should provide a good estimate of the Klason lignin content.

### Enzymatic hydrolysis

All chip samples were subjected to enzymatic hydrolysis in a custom-built metal well plate reactor described in detail elsewhere [7, 50], in which individual wells contained a total reaction mass of 450 mg. In this study, a robotics platform (Core Module, Symyx Technologies, Sunnyvale, CA, USA) loaded 4.4 mg of dry biomass into each of the wells. After biomass was loaded into all the wells in the plate, it was removed from the robot’s deck, and 435.2 μL of deionized (DI) H_2_O was pipetted into each well (8 channel pipetter, 30–300 μL, Eppendorf, Hamburg, Germany) to achieve a solid loading of approximately 1 % w/w. After allowing the biomass to soak overnight, 23.8 μL of a mixture of 1 M citric acid buffer (pH 4.95), sodium azide solution (1 g/L), and enzyme mixture was pipetted into each well (8 channel pipetter, 10–100 μL, Eppendorf, Hamburg, Germany). The mixture contained 5.227 mL of buffer, 1.045 mL of sodium azide solution, and 0.858 mL of a dilute cellulase (Spezyme^®^ CP, lot no: 3016295230, 116 mg protein/mL) and xylanase (Multifect, lot no: 301-04021-015, 56.6 mg protein/mL) (Genencor, Palo Alto, CA, USA) solution prepared at a protein mass ratio of 3:1, respectively, to which DI water was added at a volume ratio of 3:1. The enzyme loading corresponded to 30 mg cellulase + 10 mg xylanase per gram glucan in the raw material, which had a composition of 49.8 % glucan, 17.6 % xylan, and 19.8 % AcIR. After the addition of the enzyme/buffer/biocide solution, the plate assembly was sealed as described previously [7], and placed on its side in an incubation shaker (Multitron Infors-HT, ATR Biotech, Laurel, MD, USA) at 50 °C and 150 rpm. All saccharification experiments were performed in triplicate.

Replicate plates were prepared to allow sampling at different times, including 24, 48, and 168 h after incubation. At each time, the respective well plate was removed from the shaker, and the slurry from each well was transferred to 1.5 mL polypropylene (PP) centrifuge tubes (Safe-Lock 1.5 mL test tubes, Eppendorf, Hamburg, Germany) for centrifugation (5415 D, Eppendorf, Hamburg, Germany) for 5 min at 18,200 g and then 300 µL of hydrolyzate was transferred to HPLC vials for analysis.

### Magnetic resonance imaging

Chips were never frozen but were stored at 4 °C prior to conditioning. Untreated and pretreated chips were conditioned in a sealed desiccator at 25 °C and ~100 % relative humidity environment over a 0.01 (w/v) NaN_3_ solution for 14 days. Magnetic resonance images were taken on a 7 Tesla Bruker Pharmascan (300 MHz ^1^H frequency) with a Doty CP (circular polarized) rf-coil that has an interior diameter of 60 mm. During image acquisition, the samples were kept in sealed plastic bags. Zero time echo (ZTE) images [38, 40] were collected with a pulse length of 1 ms and a pulse power giving a nominal tip angle *β* of *π*/72 (5°) at the resonant frequency. With a 10-ms repetition delay, 16 averages were collected based on recording 128 data points for a 0.64 ms acquisition time. The image voxel size was ~0.5 mm, and the slice thickness was ~1.0 mm. The *T*_2_ images were collected by a multi-slice multi-echo (MSME) acquisition characterized by a pulse time of 1 ms with the pulse power giving a nominal *β* = *π*/2 (90°) at the resonant frequency. An effective TE of 7.9 ms resulted for the first echo. Eight echoes were recorded, and 64 data points were acquired at a repetition delay of 5000 ms for each echo signal from the wood sample. The image voxel size and slice thickness was ~1.0 mm.

### Simons’ stain

Direct Blue (DB, Pontamine Fast Sky Blue 6BX) and Direct Orange (DO, Pontamine Fast Orange 6RN) dyes were obtained from Pylam Products (Garden City, NY, USA). A modified version of the Simons’ staining (SS) procedure developed previously was used [46]. The fractionation of the orange dye was performed by filtering a 1 % (w/v) solution of orange dye through a 100 K ultrafiltration membrane using an Amicon ultrafiltration apparatus under 28 psi nitrogen gas pressure [47]. The orange dye solution was poured into the apparatus and filtered until 20 % of the original solution was left. 1.0 mL of the retained dye on the filter was dried in a 50 °C vacuum oven for 5 days, and the weight of the solid residue was measured to determine the concentration of the filtered solution. This result was then used to dilute the filtered orange solution to the concentration required (10 mg/mL) for Simon staining.

Next, 100 mg of biomass samples was weighed into each of five 15 mL centrifuge tubes, followed by addition of 1.0 mL of phosphate-buffered saline solution (pH 6, 0.3 M PO4, 1.4 mM NaCl) to each. Both the DB solution (10 mg/mL) and DO solution (10 mg/mL) were added in a series of increasing volumes (0.25, 0.50, 0.75, 1.0, 1.5 mL) to a series of five tubes, each containing the biomass sample and the buffer solution to create a 1:1 mixture of DO and DB dyes at increasing concentrations. DI water was then added to each tube to make the final volume 10.0 mL. All of these tubes were then incubated at 70 °C with shaking at 200 rpm for 6 h. Afterwards, the tubes were centrifuged at 10,000 rpm for 8 min to remove all of the solids. The supernatant was then placed in a cuvette, and the absorbance was read on a Lambda 35 UV–vis spectrophotometer at 455 nm and 624 nm. The concentrations of the DO and DB dyes (*C*_*O*_ and *C*_*B*_, respectively) in the supernatant were determined by solving the following two Lambert–Beer law equations for a binary mixture simultaneously [47] that were:

3 $${\text{A}}_{{455\;{\text{nm}}}} = \epsilon_{{{\text{O/455}}}} {\text{LC}}_{{\text{O}}} + {\text{ }}\epsilon_{{{\text{B/455}}}} {\text{LC}}_{{\text{B}}}$$

4 $${\text{A}}_{{{\text{624}}\;{\text{nm}}}} = \epsilon _{{{\text{O/624}}}} {\text{LC}}_{{\text{O}}} + \epsilon _{{{\text{B/624}}}} {\text{LC}}_{{\text{B}}}$$

where A is the absorption of the mixture, *ε* is the extinction coefficient, and L is the path length (in this case 1 cm). The extinction coefficients were calculated previously by preparing standard calibration curves for each dye at 455 and 624 nm. The coefficients used in this study were *ε*_O/455_ = 35.86, *ε*_B/455_ = 2.58, *ε*_*O*/624_ = 0.22 and ε_B/624_ = 15.26 L g^−1^ cm^−1^. The amount of dye adsorbed by biomass was then determined by the difference in the concentration of the initial added dye and the concentration of the dye in the supernatant. Total adsorption is determined as mg of dye per gram of biomass substrate. Accessibility is calculated by dividing the adsorption of the large orange dye by the adsorption of the small blue dye and multiplied by 100, whereas the accessible surface area is calculated by dividing the total dye adsorption by the accessibility, divided by 100.

### Scanning electron microscopy (SEM)

All cross-sectioned samples were mounted onto a stage and then coated with gold for 2 min by EM350 sputter. Images were acquired via a JEOL-1530 Thermally assisted field emission (TFE) Scanning electron microscope at 12 or 10 kV at the resolving powers indicated in the results.

## Abbreviations

SEM: Scanning electron microscope
TFE: Thermally assisted field emission
HTPH: High-throughput pretreatment and hydrolysis
AcIR: Acid insoluble residue
MRI: Magnetic resonance imaging
NMR: Nuclear magnetic resonance
ZTE: Zero echo time imaging
SPI: Single-point imaging
SPRITE: Single-point ramped imaging with *T*_1_ enhancement
RH: Relative humidity
